# Enhancing age and gender verification in OTT accounts using deep learning techniques

**DOI:** 10.3389/frai.2026.1763101

**Published:** 2026-04-07

**Authors:** M. Sanjay, Pillaram Manoj, S. Graceline Jasmine, R. Sriganth, J. L. Febin Daya, Benin Russel

**Affiliations:** 1 School of Computer Science and Engineering, Vellore Institute of Technology (VIT), Chennai, India; 2 Electric Vehicles Incubation, Testing and Research Center, Vellore Institute of Technology (VIT), Chennai, India; 3 Tata Elxsi Pvt. Ltd., Chennai, India

**Keywords:** age and gender identification, deep learning techniques, digital child safety measures, OTT (Over-The-Top), parental concerns, responsible content consumption, video recognition

## Abstract

Protecting children from inappropriate audio–visual content requires making sure they are exposed to age and gender-appropriate information. This research presents an innovative method for ascertaining user age in OTT (Over-The-Top) accounts with a tailored convolutional neural network (CNN) model, aimed at limiting access to inappropriate content. The proposed method aims to accurately evaluate the age appropriateness of content and restrict access to information that is not suitable for children, addressing the issue of children often utilizing their parents’ accounts. OTT platforms enhance their responsibilities by providing suitable content to customers through age identification, so creating a safer and more secure digital environment for all users. This solution not only promotes responsible content consumption among minors but also reduces parental concerns regarding their children’s OTT usage. The proposed model provides better accuracy of 91% for UTK Face dataset for the age and gender identification. A user interface (UI) for age verification is developed using OpenCV and Flask Framework. By addressing the crucial issue of child safety, prohibiting minors from accessing unsuitable information, and promoting responsible content consumption, the proposed solution establishes a new benchmark for OTT platforms.

## 1 Introduction

The Over-The-Top (OTT) platforms are a contemporary technological phenomenon that has gained immense popularity among the general public. It enables them to view movies, series, and videos at their preference. It is crucial to ensure that children have access to materials that are appropriate for their age and gender in order to prevent them from being exposed to inappropriate information. Reliable age verification methods within OTT accounts are required due to the growing number of children who are using their parents’ accounts to access a variety of materials. Consequently, we intend to reinforce the deep learning techniques to improve the detection of age and gender and to facilitate the responsible viewing of content by children. This not only addresses the concerns of parents regarding their children’s consumption of OTT content, but also underscores the responsibility of OTT in providing content that is acceptable to its customers.

We recommend employing the Flask Framework, OpenCV, and deep learning techniques to determine the user’s gender and age. OpenCV is equipped with a robust computer vision framework that is capable of conducting image and video analysis. It is capable of a wide range of functions, including the identification of faces, which can be employed to estimate gender and age. The interface for the system will be constructed using the Flask Framework, a lightweight web application framework. The actual determination of age and gender will be conducted using deep learning methods, specifically convolutional neural networks (CNN). CNNs have been effectively implemented in numerous computer vision applications, including gender classification and face recognition. A robust classifier that competes with other systems to provide an accurate measurement of the user’s age and gender can be constructed by utilizing a large dataset for a CNN model trained on segmented images.

The age and gender of the user are validated by the proposed system upon their login to their OTT account. Users are required to submit a current image or video of themselves in a seated position. The system will subsequently apply age and gender prediction algorithms and conduct image and video recognition techniques to obtain face features. The user’s approximated age and gender will be compared to determine if they are under the legal drinking age of 18. If they are found to be underage, appropriate measures will be implemented to restrict access to content that is not appropriate for their age group. The primary objective of this strategy is to enhance the safety and security of the online environment for all users, with a particular focus on minors. In the interim, the precise identification of user gender and age will enable OTT platforms to serve content in accordance with the necessary age restrictions, thereby substantially reducing the likelihood of minors being exposed to inappropriate content. This would subsequently alleviate parental apprehensions and gradually instruct young users on how to ingest appropriate content.

A potential solution to the age-rating content issue is the implementation of deep learning to improve the verification of age and gender for OTT accounts. A dependable solution that is developed by integrating OpenCV, Flask Framework, and deep learning techniques accurately determines the user’s age and gender. This, in turn, will result in a significantly more secure and responsible online platform for all users, with a particular emphasis on children.

The proposed problem has a sociological significance to safeguarding children from inappropriate OTT content that extends beyond individual households, touching on broader issues of media, culture, and social responsibility. Unrestricted exposure to violent, sexual, and unsuitable material can distort young viewers’ perceptions of relationships, morality, and identity formation. In the digital era, where traditional parental oversight is increasingly undermined by personalized media consumption, this creates serious concerns about the erosion of family control over children’s media experiences. The absence of effective age-verification mechanisms not only exacerbates parental anxieties but also risks normalizing harmful stereotypes, widening generational divides, and exposing vulnerable groups to content beyond their cognitive and emotional capacity. At a societal level, this raises questions of ethical accountability for OTT providers, the role of regulatory bodies, and the balance between user freedom and collective responsibility for child protection. By integrating accurate age and gender verification systems, OTT platforms can reinforce parental authority, promote healthier media consumption habits, and contribute to a safer digital ecosystem.

## 2 Background study

A more comprehensive understanding that emphasizes the identification of emotional states in individuals’ faces and takes into account their gender and age characteristics as inferred from their facial expressions, examines the impact of these factors on the emotions expressed through their faces, primarily from the literature. In the field of transfer learning, the current system implements deep learning models, including convolutional neural networks (CNN) and VGG-16, in conjunction with other machine learning techniques, including support vector machines (SVM) and K-nearest neighbors (KNN). The effectiveness of deep learning models, particularly VGG-16, for the purposes of emotion recognition, gender recognition, and age estimation has been demonstrated by the results of experimentation conducted in this manner. The examination will concentrate on the applications of these technologies in various fields, including psychology, computer vision, and interaction design.

In recent years, there has been a substantial increase in the interest in facial analysis through intelligent systems that incorporate classical machine learning with advanced deep learning. These models have demonstrated exceptional capabilities in the identification of gender, the estimation of age from facial expressions, and the detection of emotions. Their effectiveness is exemplified by models like VGG-16, which achieve exceptional accuracy in all recognition tasks. These studies underscore the significance of comprehending the interplay between emotions, gender, and age, which has practical implications for human–computer interaction, psychology, and security (Chavali et al., 2023).

Age and gender detection methodologies, which are based on image analysis, have also been applied to applications such as diet recommendation. Approaches frequently integrate SVMs for feature selection with deep convolutional neural networks (DCNNs) for feature extraction. Hybrid models consistently outperform traditional systems, attaining exceptional classification accuracy, precision, and recall rates, as evidenced by experimental comparisons against established architectures such as VGG16, VGG19, and Inception V3 (Haseena et al., 2022).

Face age and gender recognition literature encompasses numerous domains, such as consumer profiling, social media marketing, image retrieval, and image security. Although single-attribute predictions (age or gender) are extensively researched, multi-attribute predictions are more intricate due to the necessity of simultaneous optimization of gender classification and age regression. Newer architectures, such as GoogLeNet and ResNet18, outperform older models, such as AlexNet and VGG16, in terms of performance (Gupta and Nain, 2023; ElKarazle et al., 2022). Research has identified significant challenges, including data mismatch, varying aging trends, and image quality.

Convolutional neural networks are further emphasized in the context of automated age and gender prediction by research, which highlights their potential applications in online platforms, CCTV systems, and intelligence agencies. This application space is further enriched by mobile data and telecommunications studies, which illustrate the potential of age and gender classification to facilitate customer profiling and behavioral analysis (Veena and Antony, 2022; Al-Zuabi et al., 2019). In the same way, CNN-based frameworks, particularly those that employ VGGNet variations and deep CNNs have demonstrated efficacy in the face of inadequate training data, thereby bolstering their adaptability (Dhomne et al., 2018).

In the context of automated age estimation, comparative studies of deep learning architectures demonstrate that models such as Xception outperform others. Other applications include digital forensics, such as child protection investigations, in which accurate age estimation is essential. Validated on datasets such as Adience, Images of Groups, and MORPH II, complementary methods integrate CNN-based feature extraction with SVM-based classification (Othmani et al., 2020; Zaghbani et al., 2018; Ramya et al., 2022). Furthermore, the results indicate that gender can have an impact on emotion recognition, with women routinely outperforming men in the identification of nuanced expressions (Hoffmann et al., 2010).

In order to confront obstacles in facial analysis and emotion recognition, numerous sophisticated models have been proposed. These consist of hybrid CNNs, attention mechanisms, and systems that integrate preprocessing, feature extraction, and classification. Attention-enhanced CNNs have demonstrated resilience against noise and distortions, thereby enhancing the accuracy of predictions in the recognition of gender and age (He and Chen, 2021; Rodriguez et al., 2017). As a result of the scarcity of suitable datasets, the Iranian Face Database was established as a resource to enhance age classification research (Dehshibi and Bastanfard, 2010). The advantages of multi-task learning for simultaneous regression and classification are further underscored by innovative methods such as MIMO networks (Liu et al., 2023).

In addition, research investigates multimodal systems that integrate longitudinal data with CNNs to enhance the prediction of age and gender, further expanding their applications to IVR systems and voice-based services. The broader utility of service personalization is suggested by the fact that these methods reduce error rates across various demographic groups (Sanchez-Hevia et al., 2022). Bayesian classifiers and discriminative indicators are proposed in other works to enhance accuracy across age-separated datasets such as FG-NET and MORPH, with a particular emphasis on age progression and verification (Ramana and Chellappa, 2006; Mahalingam and Kambhamettu, 2012).

In parallel, the pervasive adoption of OTT platforms during the COVID-19 pandemic has sparked research that connects user behavior with demographic factors such as age and gender. Research underscores the impact of factors such as habit, trust, navigability, and hedonic motivation on the intentions to subscribe to and continue using streaming services (Soren and Chakraborty, 2023; Chen et al., 2023; Abidi and Filali, 2023; Chakraborty et al., 2023; Menon, 2022; Nagaraj et al., 2021). These observations underscore the broader socioeconomic and behavioral implications of age and gender recognition, which extend beyond technical classification.

Recent contributions also address the challenges associated with the deployment of deep-learning models for age and gender recognition in real-world environments, particularly on peripheral devices. In complex and chaotic scenarios, enhanced architectures, such as modified FairMOT with dedicated layers for age regression and gender classification, have demonstrated promising results. Lastly, studies that implement ResNet-50 with data augmentation and optimization strategies report reduced error rates and enhanced classification scores, even when training on relatively small datasets (Mahjabin et al., 2019).

In findings, the research conducted in these 25 studies indicates that the field of age and gender recognition from facial images is swiftly evolving and has a wide range of applications, including human–computer interaction, targeted marketing, forensics, and smart city systems. Although traditional techniques are consistently outperformed by deep learning methods, the trajectory of future advancements is influenced by ongoing challenges, including dataset limitations, aging variability, and contextual biases (see Table 1).

**Table 1:** Summarizing model performance across literature review.

Model	Techniques	Application focus (paper Nos)	Model (paper No)—accuracy
CNN	Deep learning, feature extraction, dropout, hybrid with RNN/Gabor, custom CNN	Emotion (24), gender (10, 22), age (1, 5), audio (16)	CNN (24)—not provided
			CNN (10)—98%
			CNN (22)—86.42%
			CNN (1)—91.17%
			CNN (5)—not provided
			CNN (16)—74%
VGG16/VGG19	Transfer learning, pre-trained models, multi-task DL, feature fusion	Emotion, age, gender (1, 2, 4, 7)	VGG16 (1)—95.3%
			VGG19 (2)—98.8%
			VGG19 (4)—Not provided
			VGG19 (7)—95%
SVM	Hybrid DL + ML, HPSO optimization, feature-based classification	Emotion (12), Age (1)	SVM (12)—92%
			SVM (1)—66.78%
ResNet	Transfer learning, residual learning, comparative DL	Age estimation (4)	ResNet (4)—not provided
GoogLeNet	Deep transfer learning	Age estimation (4)	GoogLeNet (4)—not provided
AlexNet	Pre-trained deep learning	Age estimation (4)	AlexNet (4)—not provided
RNN/LSTM	Sequential modelling, audio classification	Age/gender (16)	RNN/LSTM (16)—76%
Xception	Comparative deep learning models	Age estimation (8)	Xception (8)—MAE (20.1)
Autoencoders	Deep embedding learning, latent feature encoding	Age estimation in forensics (9)	Autoencoders (9)—MAE (3.34)
Bayesian classifier	Probabilistic modelling, identity verification	Age progression (17)	Bayesian classifier (17)—error (8.5)
LBP (local binary patterns)	Hierarchical pattern analysis	Age progression (18)	LBP (18)—not provided
Attention mechanisms	Patch attention, multi-task DL	Age, gender estimation (13)	Attention mechanisms (13)—not provided
Traditional ML	KNN, decision trees, custom dataset	Age/gender (6, 14), emotion (11)	Traditional ML (6)—KNN-72%
			Traditional ML (14)—86.64%
			Traditional ML (11)—not provided
MIMO network	Multi-task Learning	Age, gender, race prediction (15)	MIMO network (15)—not provided
Gabor + CNN	Hybrid deep learning, texture filtering	Facial emotion analysis (25)	CNN (25)—MAE (<1)
VGGFace	Face embedding + SVM	Age recognition (21)	VGGFace (21)—65%

### 2.1 Research gap identified

Existing research in age and gender prediction mainly uses handcrafted features and facial landmarks and texture descriptors and deep learning–based feature extraction methods to improve classification accuracy. The majority of research studies assess their results through standard datasets while they focus on comparing different models and testing optimization methods and developing new features. The existing research works restrict their scope to offline prediction tasks while doing not show any solutions for real-world content regulation systems that need to be deployed.

The existing research fails to study how real-time age verification will work with OTT platforms to create automatic systems that restrict access to age-restricted materials. The existing research lacks studies that create a complete system which combines live camera detection with user interface elements and automatic access restriction systems. The current methods estimate user demographics instead of implementing enforcement procedures based on specific applications.

## 3 Methodology

### 3.1 Data description

The UTKFace dataset is a vast collection of photographs of faces that are well-suited for vision applications. This dataset contains nearly 20,000 photographs of individuals between the ages of 0 and 116, with pertinent identifiers for nationality, gender, and age. The collection’s diversity is apparent in the images’ position, face expressions, illumination, occlusions, resolution, and other aspects. The UTKFace dataset is well-suited for a diverse range of facial recognition, analysis, and landmark localization tasks, such as face identification and age estimation, as well as age progression and regression. Taking advantage of the openness and authenticity of this dataset in representing facial complexities across various ages, genders, and groups of people, the richness and diversity of the data from UTKFace would be utilized to develop, train, and evaluate algorithms for various facial analysis tasks.

### 3.2 Proposed system

**Figure 1:**
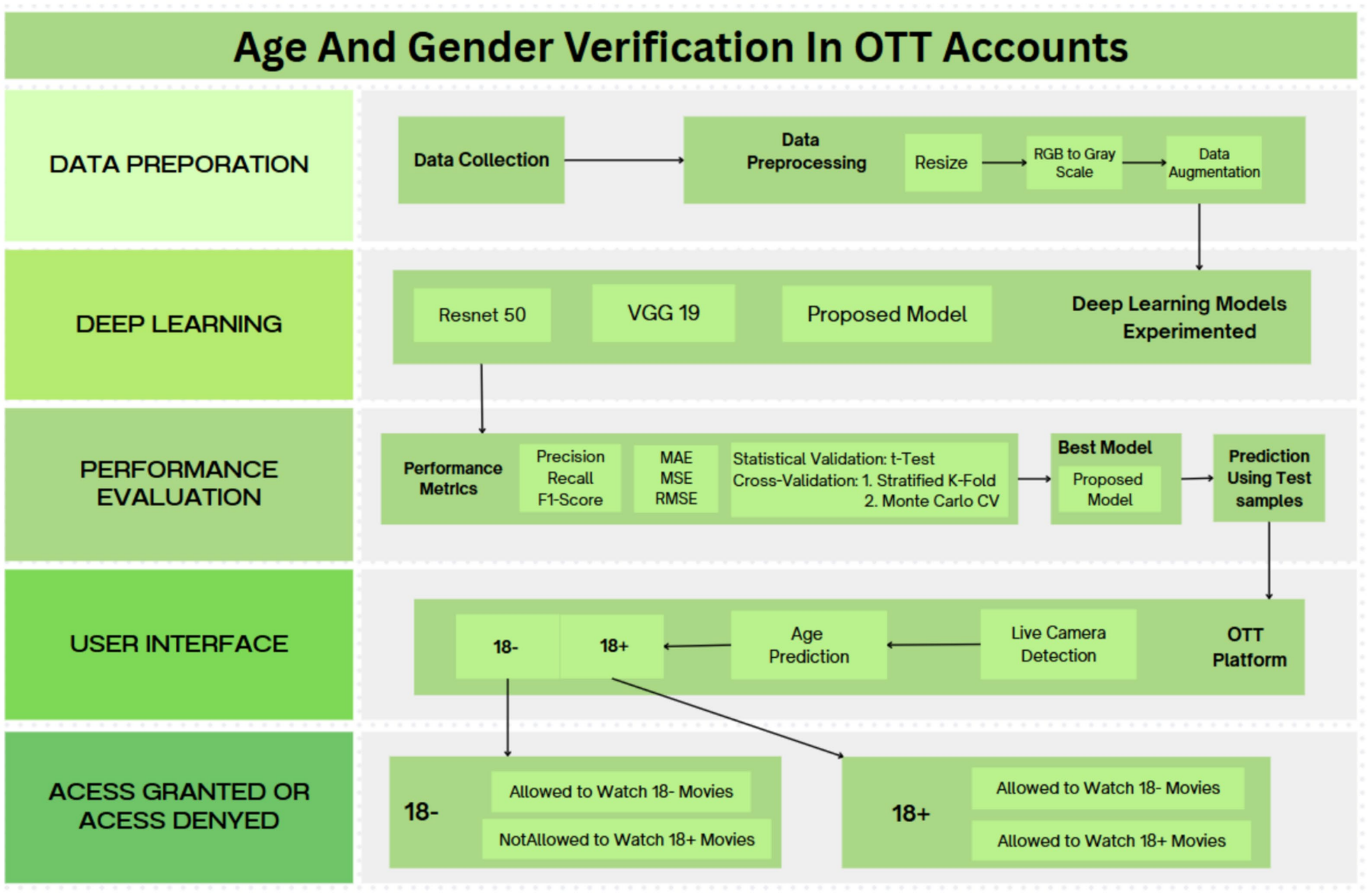
Proposed architecture of age and gender verification in OTT accounts in user interface.

Figure 1 illustrates the architecture of the proposed approach, which suggests that the age verification process for an OTT (Over-The-Top) platform. The initial step in the process is to determine whether the user is 18 years of age or older (18+ or 18−) by employing a specific technique, such as facial recognition, to determine age. In order to enhance its accuracy, the system encourages the user to upload a live photo or video if it is unable to confidently determine age. The image data undergoes normalization, grayscale conversion, and certain data augmentation procedures, including image rotation, resizing, inverting, and cropping, during input preprocessing. Color jittering is the process of enhancing the luminance, contrast, saturation, and hue of a generalization. Additionally, these modifications facilitate the model’s generalization to a variety of input conditions. These augmented datasets are employed to train a deep learning model that is customized to analyze user age and gender. The model is composed of CNN, VGG19, and ResNet50. Metric parameters were implemented to evaluate the performance of the models. These augmented datasets are used to train deep learning models, including Customized CNN, VGG19, and ResNet50, in order to accurately analyze user age and gender. Key metrics, including MAE, MSE, RMSE, Precision, Recall, F1-Score, Cross-Validation: Stratified K-Fold, Monte Carlo CV, and Statistical Validation: *t*-Test, were employed to assess the performance of the model. The Customized CNN model demonstrated the most exceptional overall performance of the three, with ResNet50 following closely behind. On the other hand, VGG19 demonstrated inferior generalization capabilities, particularly in the areas of Recall, F1-Score, Cross-Validation, and Statistical Validation.

OpenCV serves face detection and region cropping functions within the proposed framework while it does not support handcrafted feature extraction. The deep learning models implement feature extraction through their convolutional layers which create hierarchical representations from input pixel data (Customized CNN, ResNet50, VGG19). The system operates without using facial landmarks or geometric face features which require manual development. The training process did not include masked faces as part of its training data. The presence of masks decreases prediction accuracy because they block essential facial features. The current system cannot process masked faces which we plan to improve through specific data enhancement techniques and training methods in upcoming research.

### 3.3 User interface

Essentially, it is a user interface that enables the user to conduct a live age verification and validate the model based on the accuracy of its age predictions. As per the model’s prediction, the viewer will be permitted to view movies with an 18+ rating, restricted from viewing any movies with a classification below 18, or able to watch all types of movies, including those under 18. The system as a whole integrates technologies such as deep learning and face recognition, as well as information provided by users, to ensure that the age of the content is accurate. It also denies access to age-inappropriate content to individuals who are not of the mission age and provides content that is viewable without restriction to all audiences. In order to ensure that the OTT platform offers a safe and age-appropriate viewing experience, these age verification systems are necessary for the maintenance of content rules. It will provide the user with a user interface that allows them to capture a live photograph or video for the purpose of verifying their age.

## 4 Model description

### 4.1 Customized CNN

**Figure 2:**
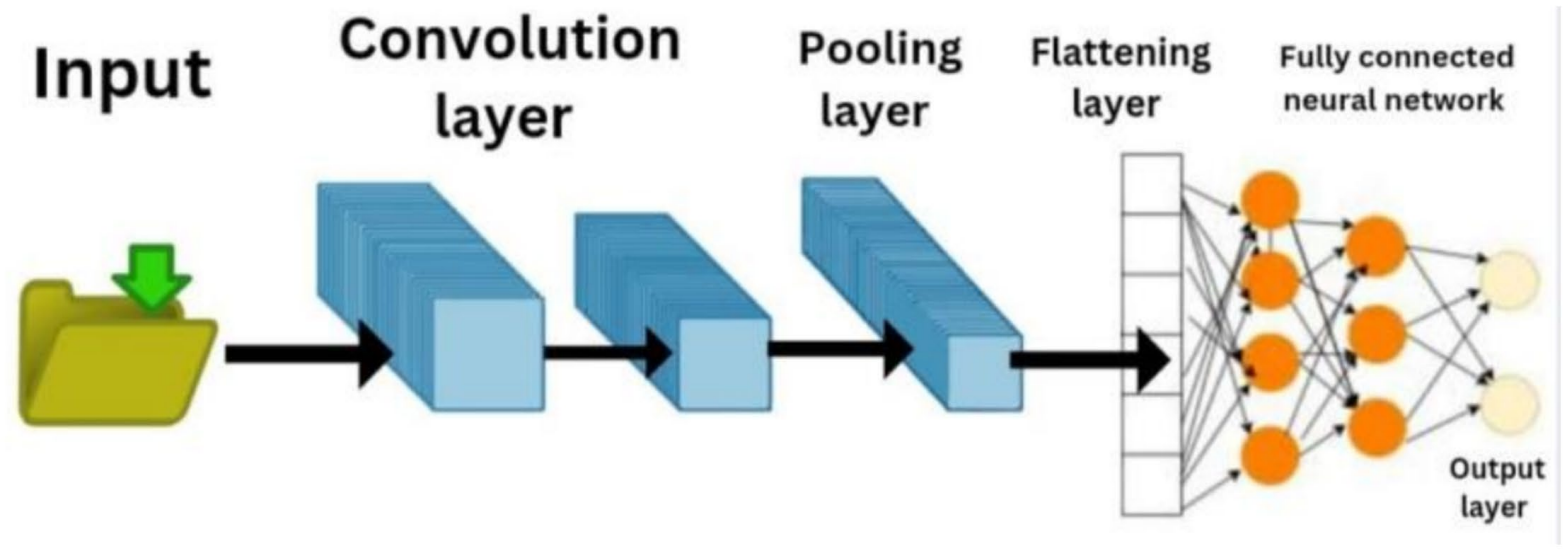
Architecture for Customized CNN model.

The architecture of customized convolutional neural networks (CNN) is illustrated in Figure 2. It uses multiple convolutional and pooling layers to build up its system before reaching its final classification stage through fully connected layers. The input image first undergoes processing through multiple Conv2D layers which use 3 × 3 kernels and ReLU activation to extract its spatial features at different levels. The Max Pooling layer which uses 2 × 2 dimensions follows each convolutional block to maintain important features while decreasing the spatial size of the data. The system first extracts features from the data before it transforms the output into fully connected dense layers through a process of flattening. A Dropout layer (rate = 0.5) is incorporated before the final classification layer to reduce overfitting. The final output layer uses Softmax activation for age and gender classification. The model trains from its initial state because it needs to develop its specific task features which will come from the data set. Customized CNN have been well appreciated concerning their capability to capture spatial hierarchies, and they have proven to be very probably successful in image analysis and recognition tasks.

### 4.2 ResNet50

**Figure 3:**
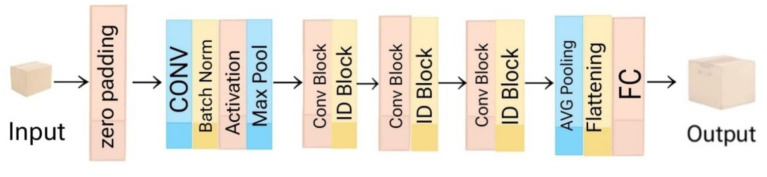
Architecture for ResNet50 model.

Figure 3 represents the architecture of ResNet50 model. It is a deep residual learning network which contains 50 layers uses residual connections to achieve effective gradient movement while avoiding the vanishing gradient issue. The model starts with a convolutional layer which is followed by Batch Normalization and ReLU activation together with a 3 × 3 Max Pooling layer that performs initial spatial downsampling. The system consists of multiple residual blocks which use 3 × 3 convolutional kernels together with identity mappings. The model uses Global Average Pooling as the replacement for traditional flattening which occurs after convolution before entering the fully connected classification layer. The researchers use a pre-trained ResNet50 model which they initialized with ImageNet weights through transfer learning. The final dense layers were changed to enable age and gender classification while specific layers were optimized to increase overall system performance.

### 4.3 VGG19

**Figure 4:**
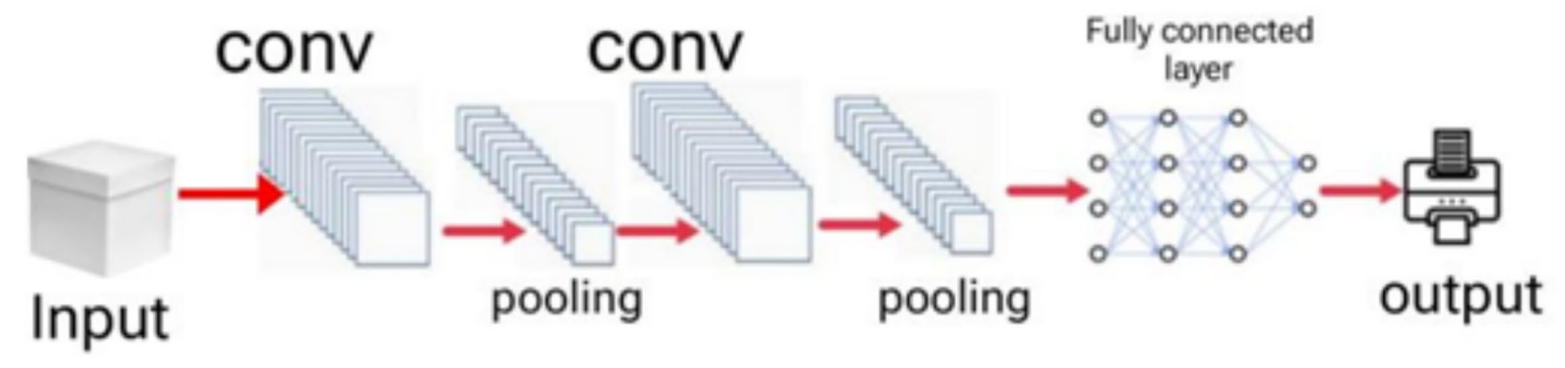
Architecture for VGG19 model.

Figure 4 represents the architecture of VGG19 model. It is a deep convolutional neural network that operates through 16 convolutional layers and 3 fully connected layers. The convolutional layers of the network use 3 × 3 kernels which all use ReLU activation as their activation function. The system uses a 2 × 2 Max Pooling layer after each convolutional block to decrease its spatial dimensions. VGG19 operates with a continuous processing flow because it does not implement residual connections which distinguishes it from ResNet50. The study begins with ImageNet pre-trained weights to establish the convolutional base while it customizes the final fully connected layers for age and gender prediction. The model uses transfer learning to take advantage of existing visual features which it combines with new features to support its classification task.

## 5 Results and discussion

### 5.1 Customized CNN

**Figure 5:**
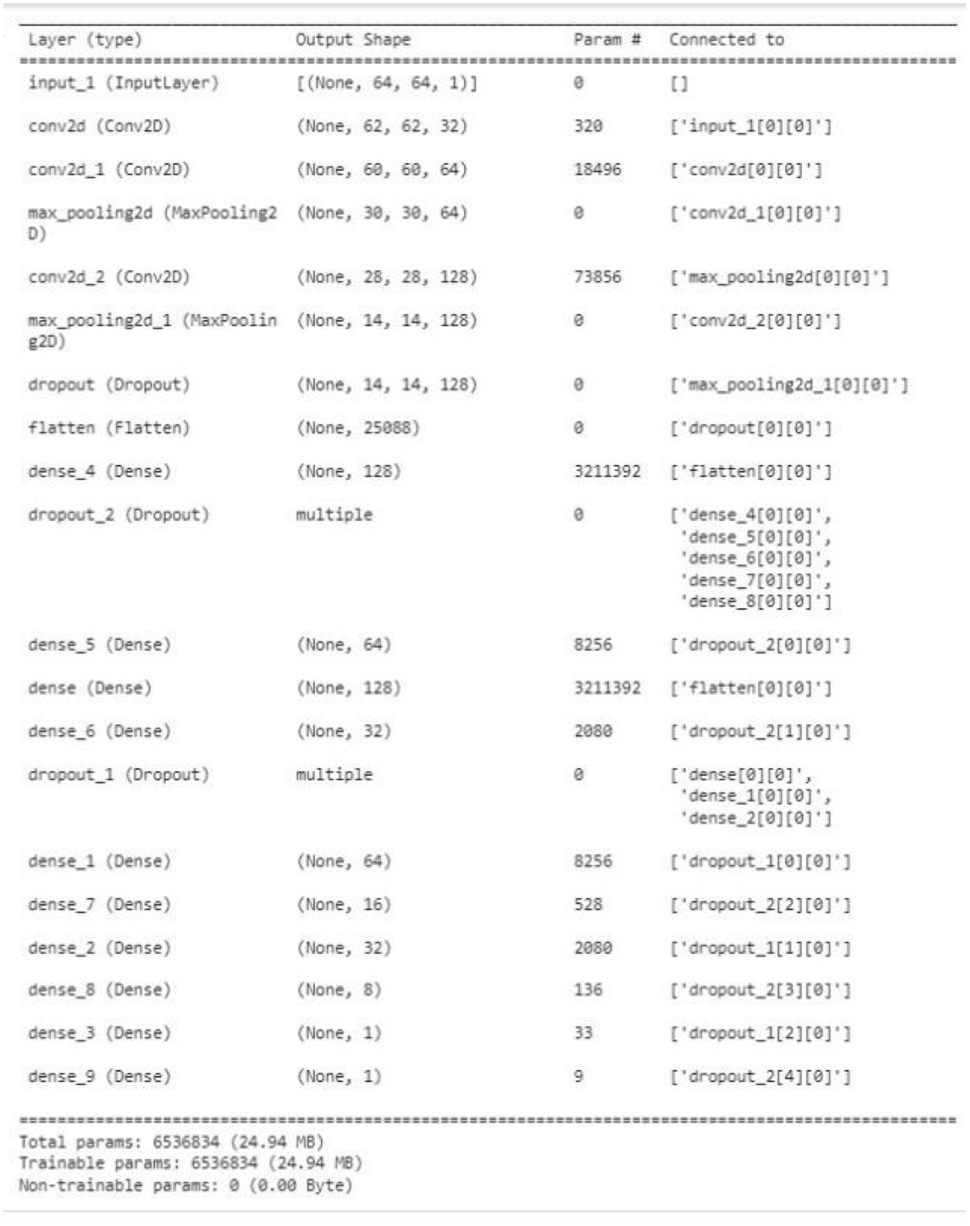
Model summary of Customized CNN algorithm.

The summary of the Customized CNN algorithm is illustrated in Figure 5. The Customized CNN model consists of ten layers, including two convolutional layers, two pooling layers, and six fully connected layers. This results in a total of 6,536,834 trainable parameters. The accuracy of the training on the provided data is 92.03%, with a training loss of 0.2277. During validation, the accuracy marginally decreased to 90.49%, with a validation loss of 0.3004. This implies that the model has acquired the ability to accurately estimate age based on the training data. However, it may have a slightly lower validation accuracy, which is indicative of its overfitting of the training data.

To improve performance, one can either continue to accumulate additional training data, concentrating on data that is similar to that of the validation set, or employ regularizing techniques (L1 or L2) to prevent overfitting or investigate a more compact model architecture. However, the Customized CNN model exhibits considerable potential in age estimation, despite the fact that it necessitates some additional refinements, as evidenced by its training performance.

**Figure 6:**
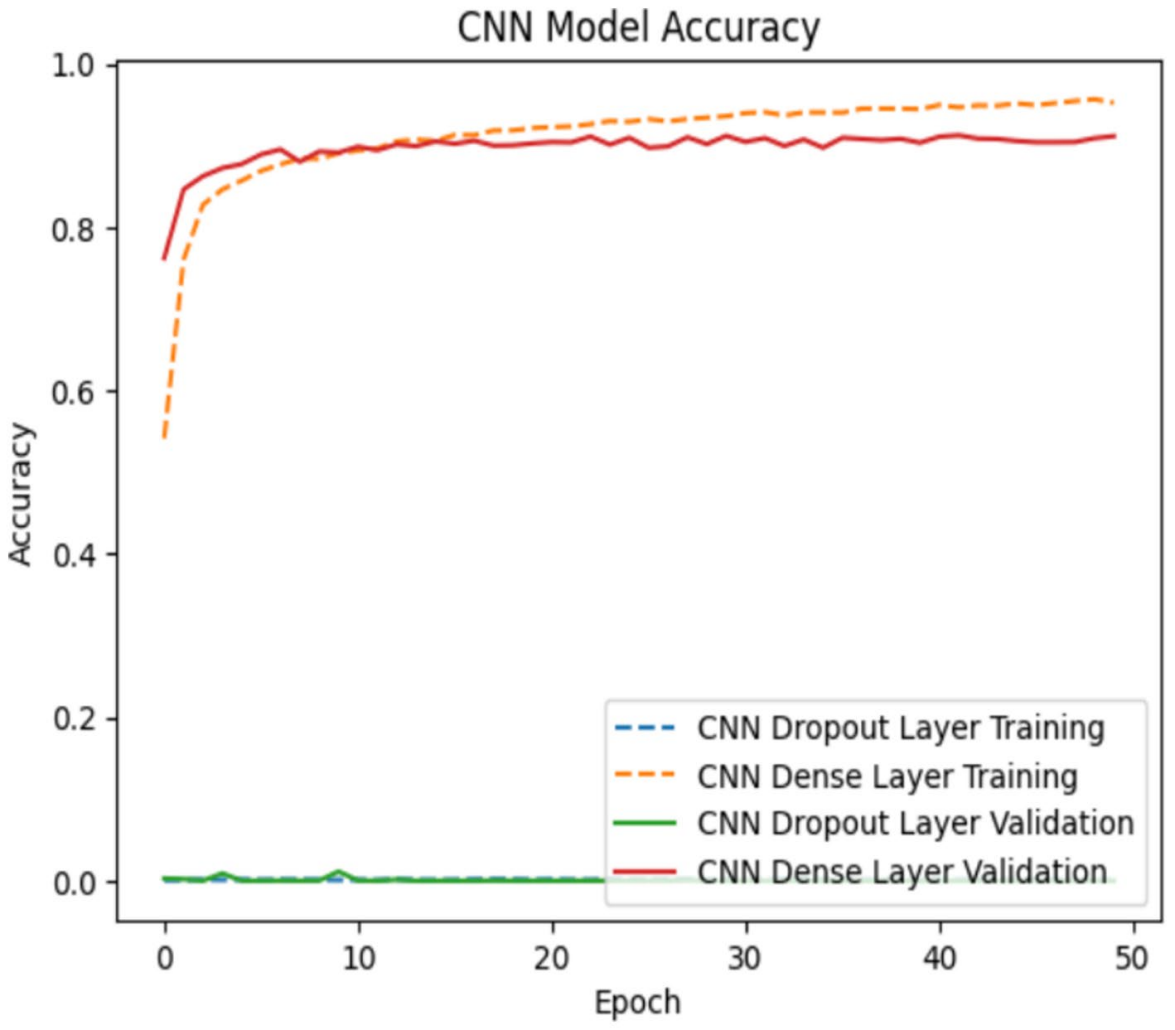
Model accuracy for Customized CNN model.

**Figure 7:**
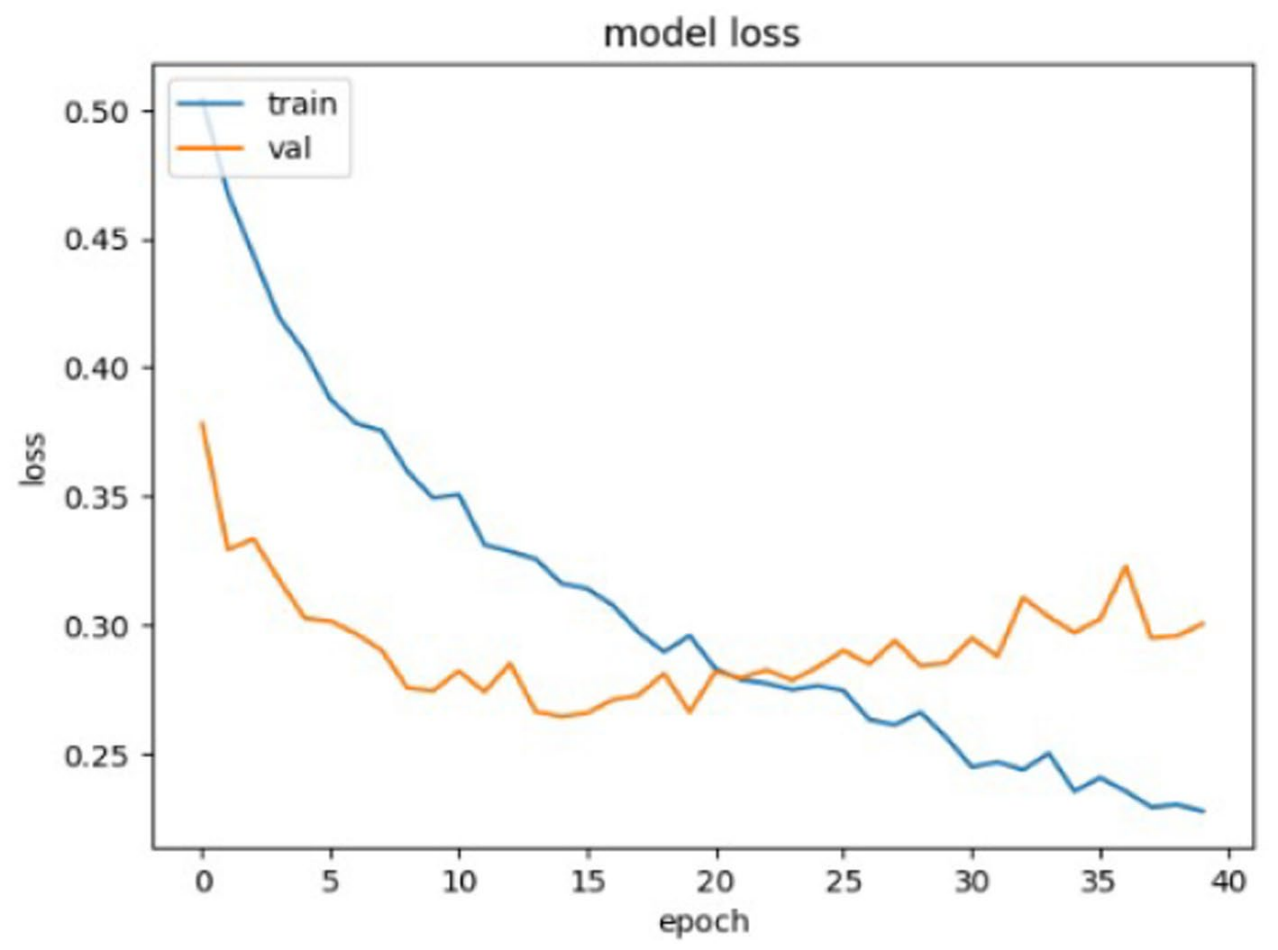
Model loss for Customized CNN model.

The Customized CNN model’s accuracy and loss determination are illustrated in Figures 6, 7. The accuracy and loss of a Customized CNN for a neural network-based deep learning model are depicted in the graph above. The loss values are displayed on the *y*-axis, while the number of training epochs is represented on the *x*-axis. The “train” curve typically denotes training losses, while the “val” curve represents validation losses. Training is conducted to gain a more comprehensive comprehension of the changes in training and validation losses. Initially, both curves may decrease as the model learns and responds to the training data. The primary objective is to commence monitoring at the moment when the training loss is decreasing but the validation loss reaches a plateau or begins to increase. This is a characteristic of overfitting, which results in the model being overly tailored to the training set and having little ability to generalize to unknown data.

The model is likely overfitting if the training loss is consistently lower than the validation loss. Consequently, it is imperative to either enhance the architecture or implement regularization measures. If both losses are substantial, it suggests underfitting, which may necessitate a more intricate model or additional training data. It is a beneficial visualization for practitioners, as it demonstrates the model’s optimization, even if the model is learning and generalizing well to data that has not yet been observed.

The training curves in Figure 7 show that the customized CNN system experiences mild overfitting problems because its architectural design implements dropout layers. The system used dropout to decrease neuron co-adaptation which should enhance generalization abilities but the system still suffers from overfitting because its dataset size and model complexity exceed current limits. The results demonstrate that regularization achieved partial success because the system requires further adjustments to achieve better performance. The next system updates will include two improvements which involve changing dropout rates and adding stronger L2 regularization and early stopping methods to improve generalization performance.

### 5.2 ResNet50

**Figure 8:**
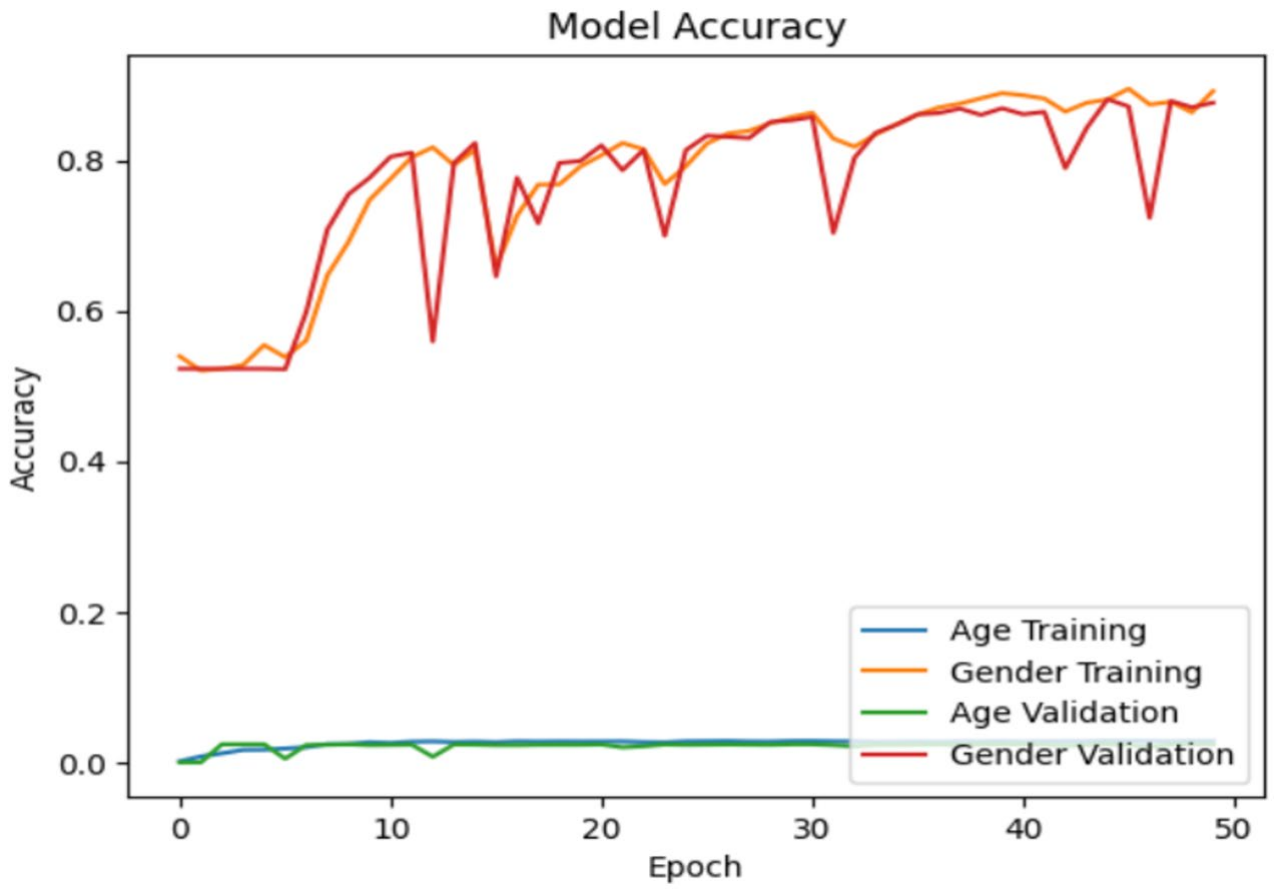
Model accuracy in ResNet50.

**Figure 9:**
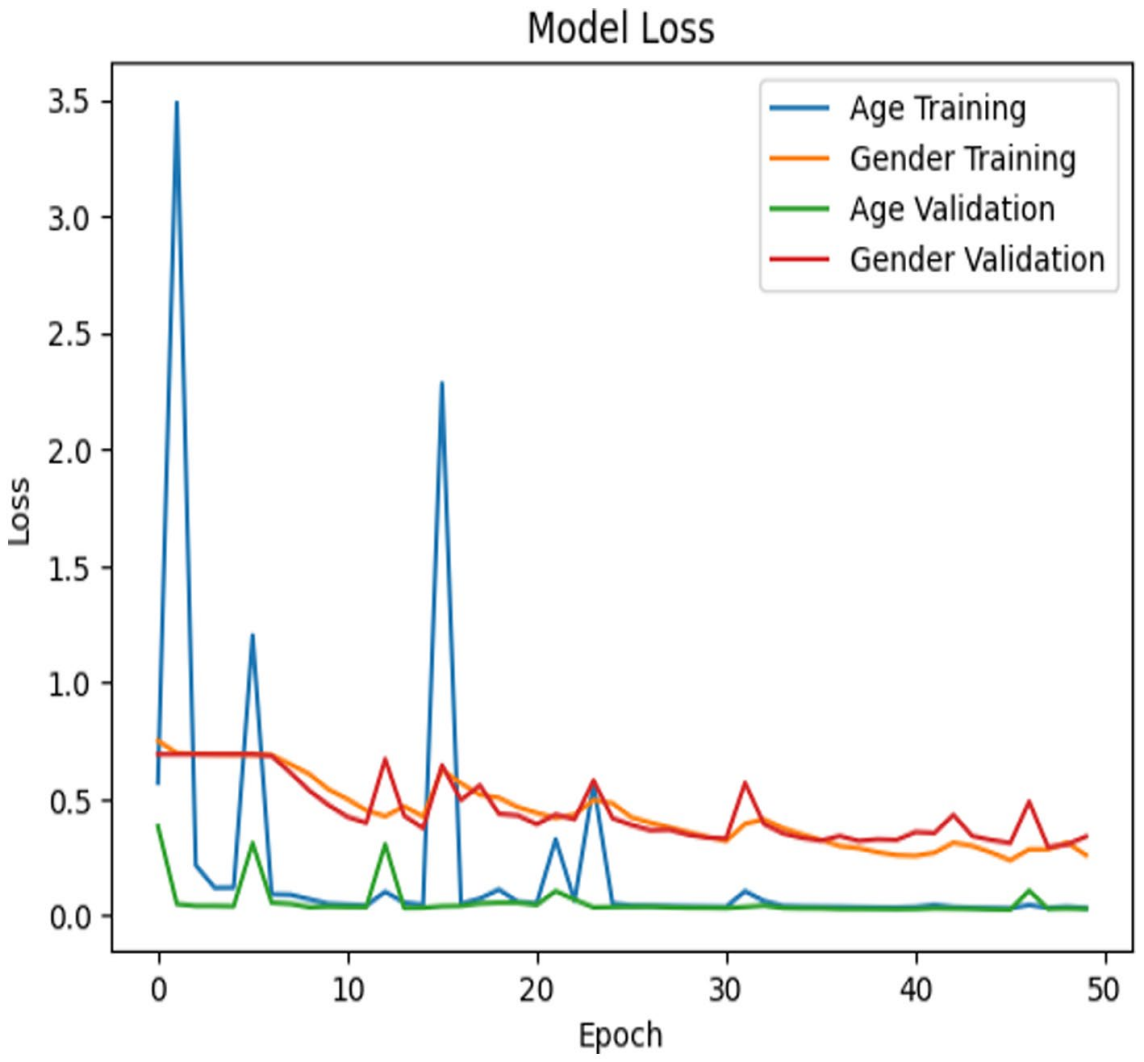
Model loss in ResNet50.

The ResNet50 model’s accuracy and loss will be illustrated in Figures 8, 9. A model’s accuracy of 0.82 indicates that it could accurately classify 82% of the data. The Adam optimizer was employed by the ResNet50 model, with loss functions that were specifically designed for the age (mean squared error) and gender (binary cross-entropy) metrics, with an emphasis on accuracy. There were 50 epochs of training, with a sample size of 128, and the accuracies and losses for the age and gender outputs were plotted separately. The accuracy plots demonstrate the model’s proficiency in classification, while the loss plots demonstrate its effectiveness in minimizing the discrepancy between its predicted and actual values. These plots serve as external indicators of the models’ training status and provide indications of overfitting or underfitting. Therefore, in order to enhance the ResNet50 model’s efficacy on this dataset, it will be necessary to experiment with alternative models or to integrate the parameters.

### 5.3 VGG19

**Figure 10:**
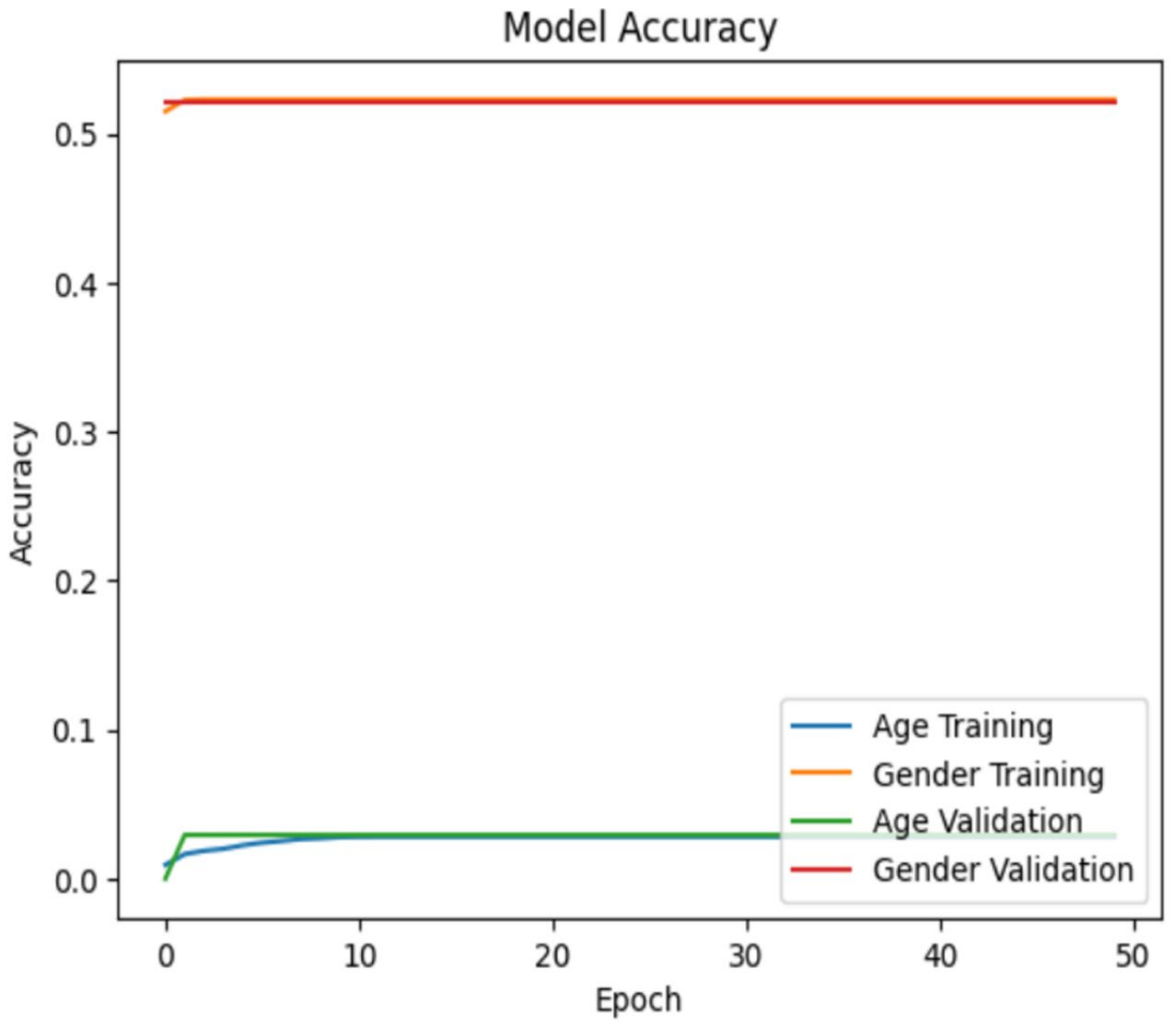
Model accuracy in VGG19.

**Figure 11:**
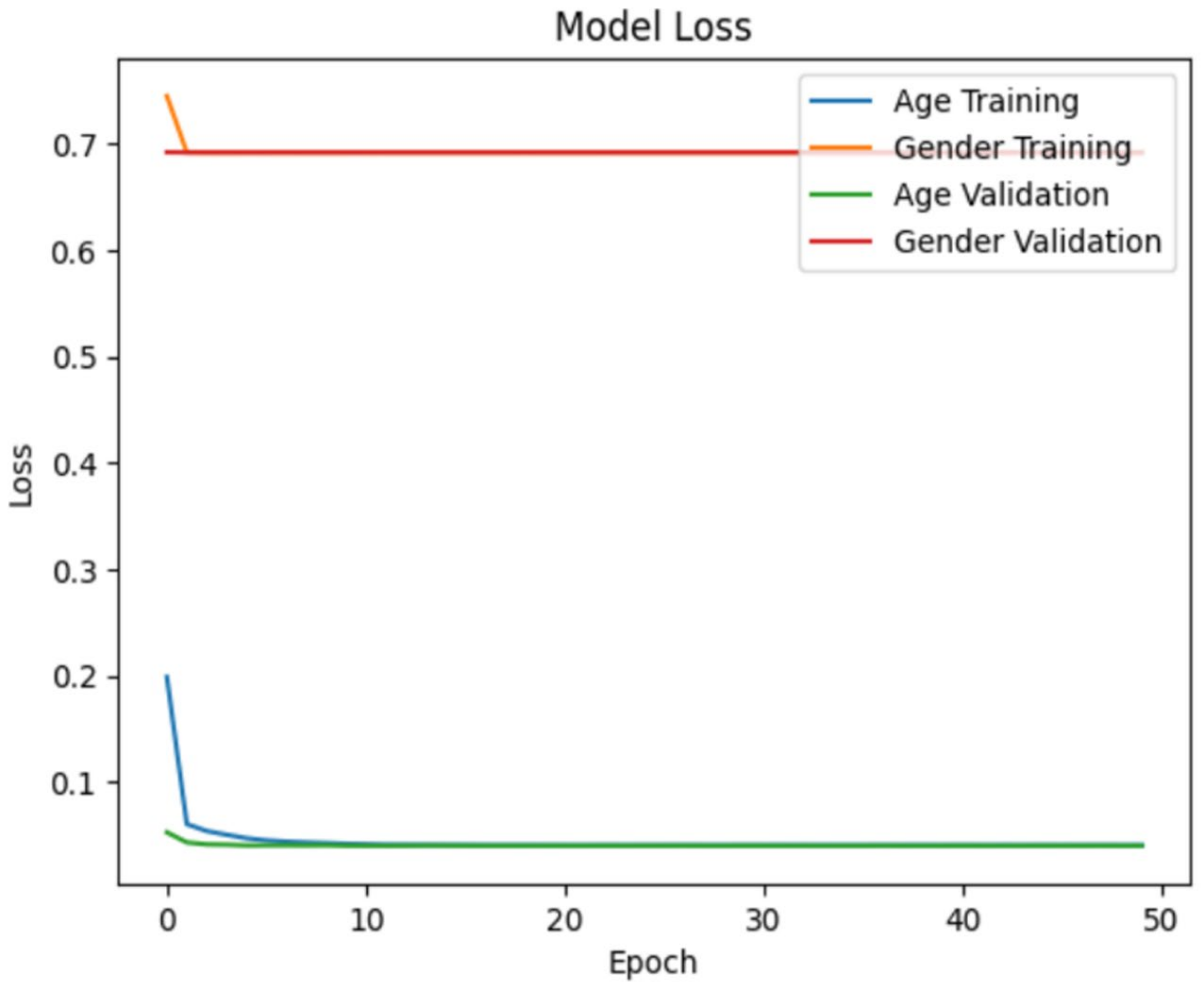
Model loss in VGG19.

The accuracy and loss of the VGG19 model are illustrated in Figures 10, 11. This is a neural network that has been trained for multi-task learning to predict age and gender simultaneously, based on VGG19. The model has correctly identified over 82% of the data, as evidenced by its accuracy of 0.82. The fundamental model, VGG19, has been pretrained on ImageNet and additionally includes custom layers for the age and gender components. The model is trained using the Adam optimizer, mean squared error loss for age prediction, and binary cross-entropy loss for gender prediction. The training and validation losses and accuracies for age and gender outputs are recorded and exhibited separately for 50 epochs and a batch size of 128. This subsequently enables the model to be evaluated in terms of its ability to minimize age prediction errors and perform more precisely with respect to gender classification during training. Loss is comparable to a function in the deep learning domain; however, it represents the aggregate of all errors that the machine generates during training and validation, as opposed to accuracy, which is quantifiable. In order to mitigate loss, it is possible to modify any hyperparameter of the deep learning model, including the collection size of whole images used for training or the number of epochs.

All three models such as Customized CNN, ResNet50, and VGG19 were trained for both age and gender classification using the same dataset and evaluation protocol. We applied transfer learning to ResNet50 and VGG19 by using pre-trained weights as initial parameters while they locked certain convolutional layers and trained only the final classification layers. The performance differences shown in Figures 7, 9, 11 result from different architectural depths and feature extraction abilities and optimization methods which were used during testing.

### 5.4 Comparison of all model accuracy’s

**Figure 12:**
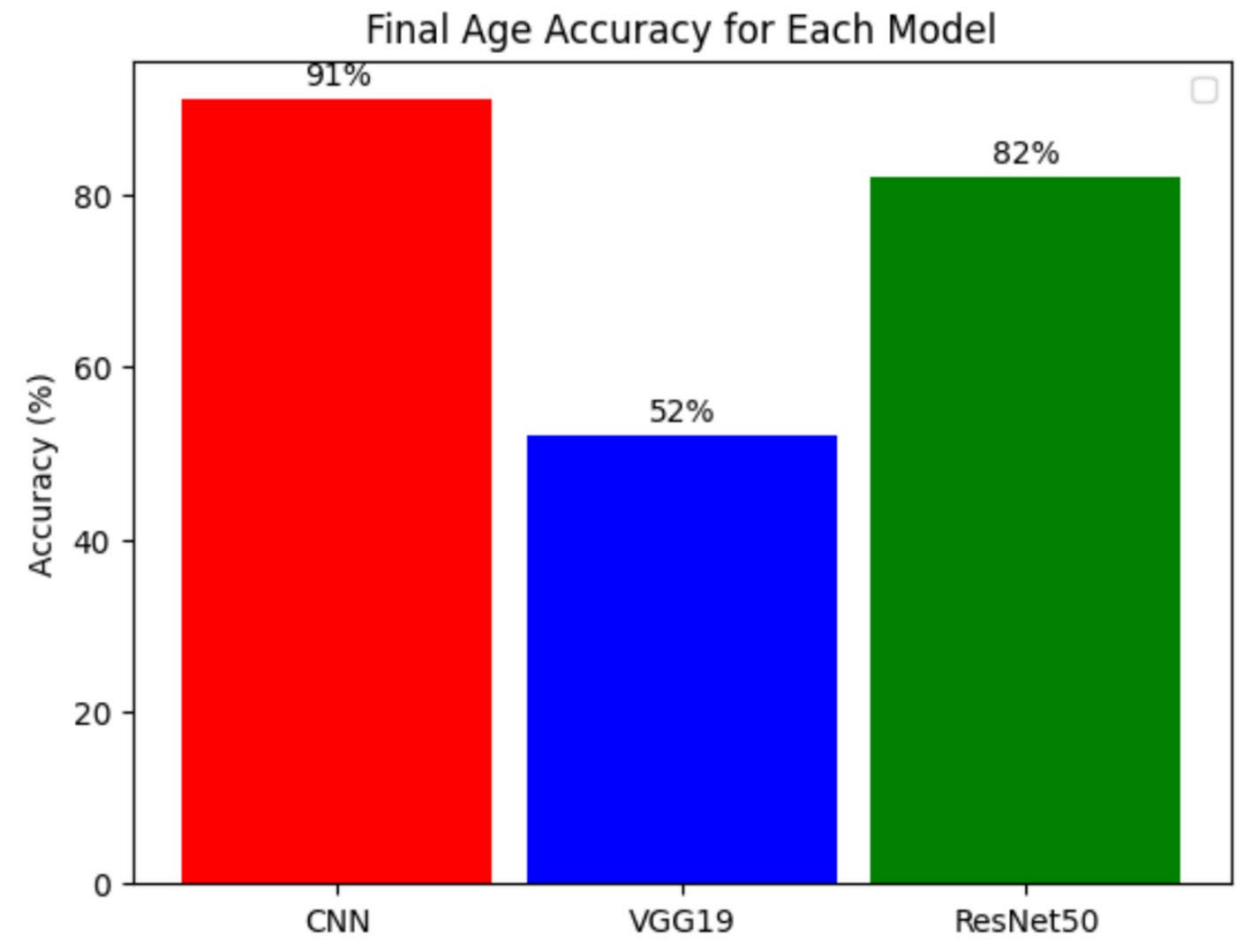
Bar chart of the final age accuracies for each model.

Figure 12 illustrates the bar chart of the final age accuracies for models including Basic CNN, VGG19, and ResNet50. Performance evaluation trials are currently underway for these three models. Customized CNN demonstrated the highest accuracy, as it is adept at learning spatial structures and effectively manages convoluted interactions with data, with a maximum cut-off of 91%. ResNet50, the second-best-performing model, achieved an accuracy of 82% due to its residual connection, which maintained information across layers. This reduced the issue of vanishing gradients and enabled the model to learn more abstract or deeper features more efficiently. This may have been due to overfitting or feature-extraction limitations, as VGG19 demonstrated a subpar performance with 52% accuracy. This suggests that architectural tuning and hyperparameter settings are necessary. The results regarding the differences in accuracy indicate that model selection and architectural design are essential factors in determining the capabilities and limitations of each design, which in turn determine performance. The performance of these models in actual work would be enhanced by future optimization and tailored architecture adjustments.

### 5.5 Performance metrics

**Figure 13:**
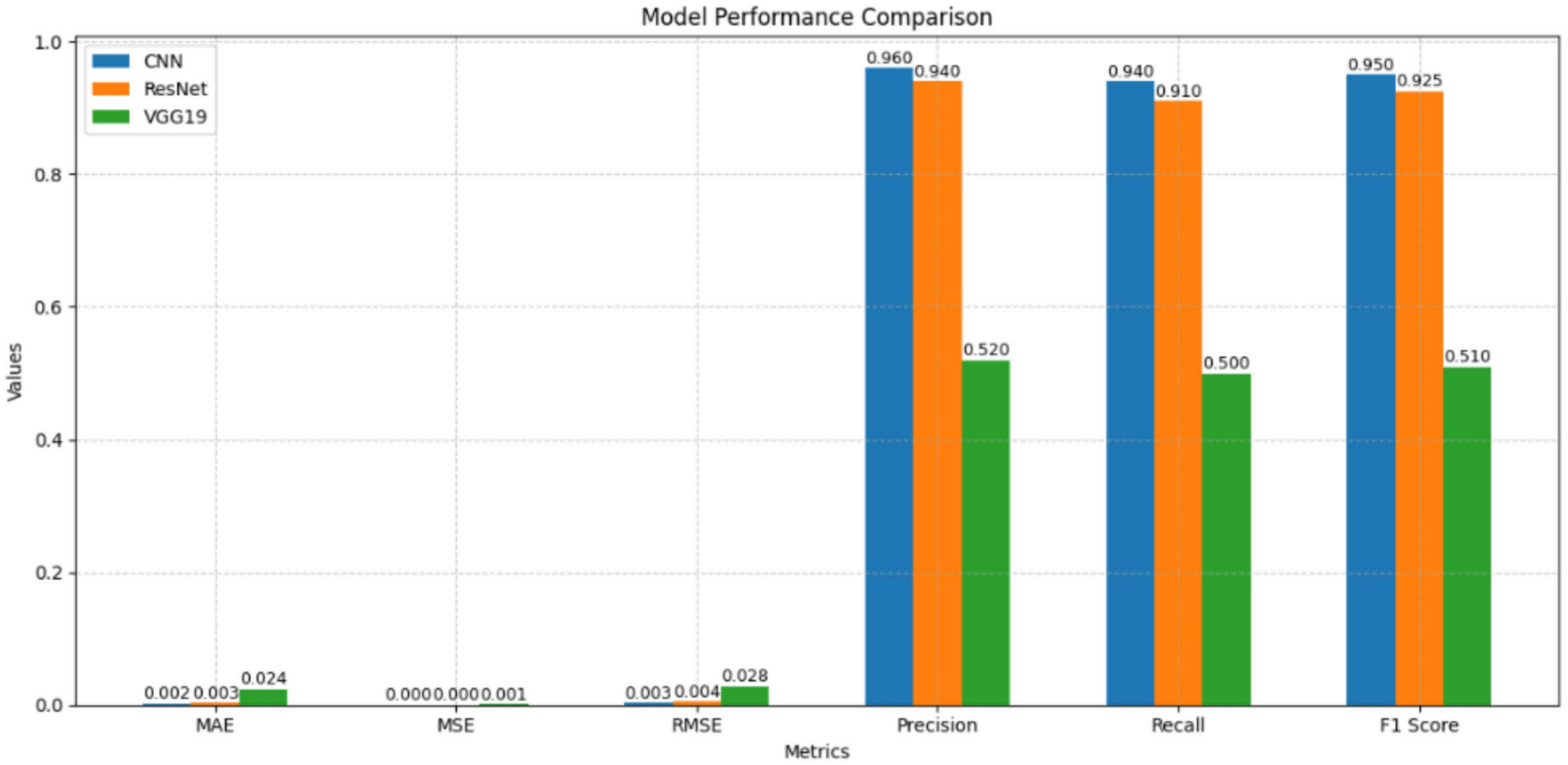
Performance metrics for all models.

The purpose of Figure 13 is to compare the three available models, namely CNN, ResNet50, and VGG19, in terms of six distinct evaluation metrics: MAE, MSE, RMSE, Precision, Recall, and F1-Score. This is achieved through the use of a bar chart. Our Customized CNN achieved the highest scores in classification (Precision-0.960, Recall-0.940, and F1-Score-0.950), as well as the least error values (MAE = 0.002, MSE = 0.000, RMSE = 0.003), thereby demonstrating good accuracy and a balanced prediction process. The ResNet50 model was the second-place finisher, with marginally exacerbated errors (MAE: 0.003, MSE: 0.001, RMSE: 0.004) but commendable classification values (Precision: 0.940, Recall: 0.910, F1-Score: 0.925). In contrast, the VGG19 is highly susceptible to disappointment due to its subpar classification values (Precision: 0.520, Recall: 0.500, F1-Score: 0.510) and elevated error rates (MAE: 0.024, MSE: 0.001, RMSE: 0.028). Consequently, it is rendered ineffectual in generalizations and predictions.

Although Precision and Recall measure different aspects of model performance, their relationship between these two metrics does not show an inverse connection because F1-Score functions as their harmonic mean which demonstrates their existing relationship. In age verification systems designed to protect children both false positive results which incorrectly identify minors as adults and false negative results which miss detecting underage users, present serious dangers. Therefore, the F1-Score is considered the most suitable primary evaluation metric, as it ensures balanced and reliable performance for secure OTT content access.

**Figure 14:**
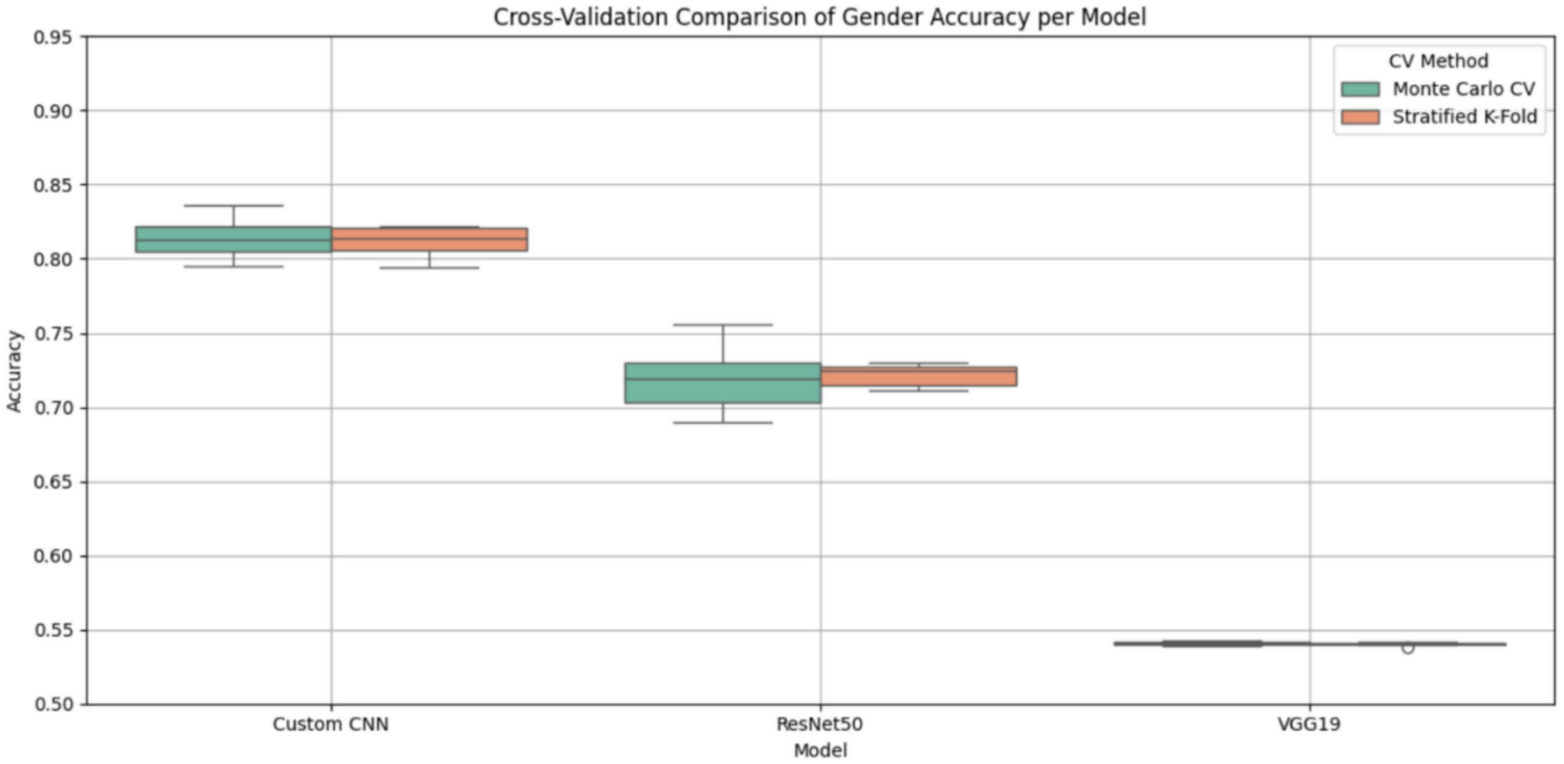
Box plot for cross-validation comparison of gender classification accuracy’s of DL models.

The cross-validation comparison of gender classification accuracy across the three deep learning models (Custom CNN, ResNet50, and VGG19) is illustrated in Figure 14, which includes Monte Carlo Cross-Validation and Stratified-K Means Cross Validation. In terms of highest median accuracy (~82%) and least variance in either case, the Custom CNN has relatively outperformed others, indicating good generalization and consistent performances. The average accuracies of ResNet50 are approximately 72–73 percent, with a significant variation under Monte Carlo CV. This suggests that the model is susceptible to overfitting and data divides. Underfitting and architectural incompatibility with the task at hand are indicated by the consistently subpar performance of VGG19 (~54%). Monte Carlo CV eliminates layers in favor of assessing robustness through a more diverse set of random splits, whereas Stratified K-Fold guarantees that the class distribution remains intact. Consequently, it would be a reliable method in this instance, given the imbalanced nature of datasets such as gender classification. The most viable and stable model for deployment is Custom CNN, which has been evaluated on both methods. However, a more comprehensive view of performance is achieved by combining the two methods.

**Figure 15:**
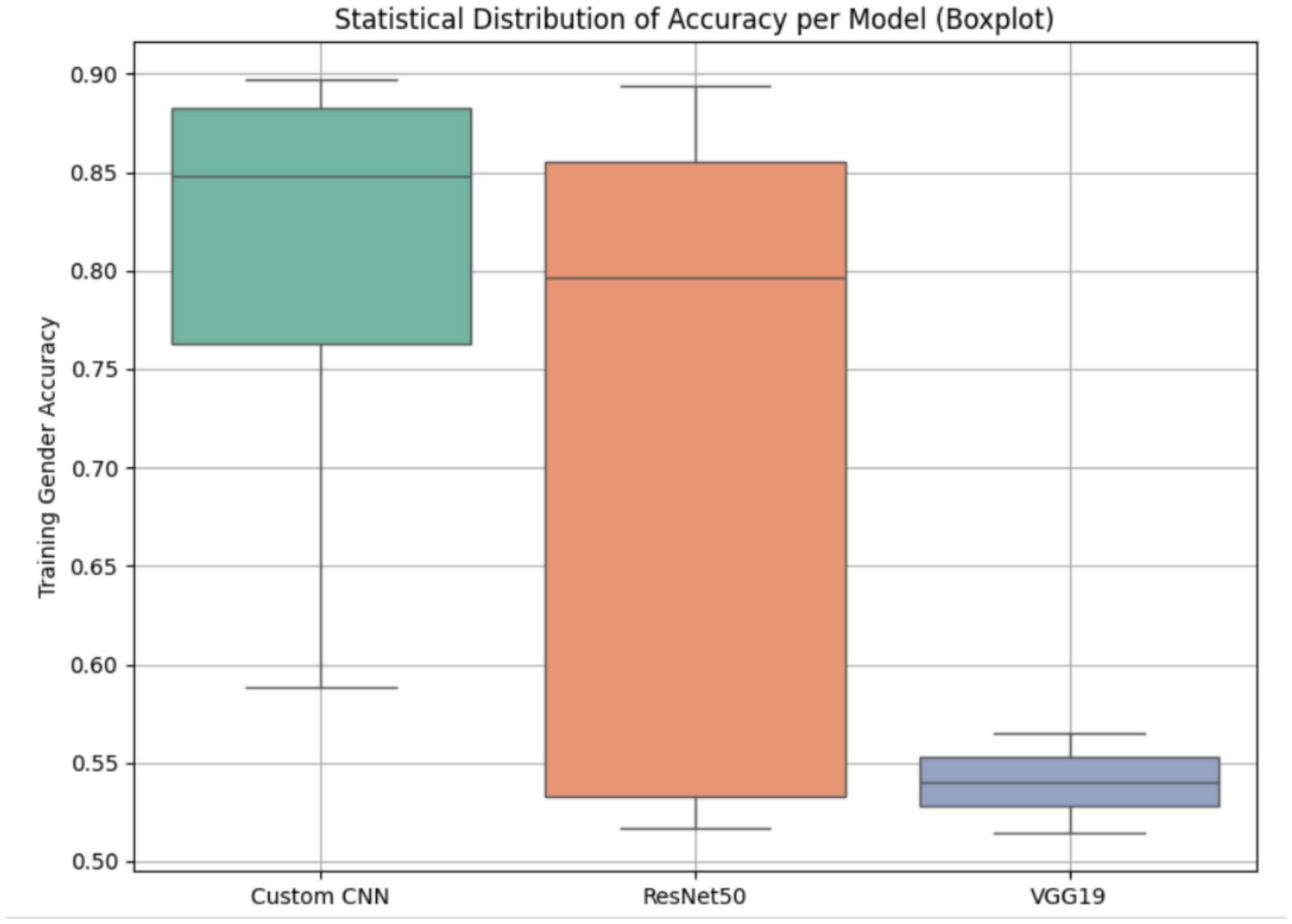
Box plot for statistical distribution with accuracy’s of DL models.

The statistical distribution of training gender classification accuracies for three deep learning models—Custom CNN, ResNet50, and VGG19—is illustrated in Figure 15 through boxplots. Highest and most stable behavior is exhibited by the Custom CNN model: Its median accuracy is nearly 0.85, and the interquartile range (IQR) is both modest and low in variance. This indicates that the model has generalized well and that the training process has been seamless. In terms of peak accuracy, ResNet50 never truly loses to its competitors. However, it exhibits a wide IQR (ranging from approximately 0.52 to 0.89), indicating significant dispersion and instability across epochs. VGG19 is a stable competitor with a minimum median accuracy level of approximately 0.54 and very minor dispersion, despite being a poor competitor. The Custom CNN was statistically confirmed to be superior to ResNet50 and VGG19 (*p* < 0.05) through a paired t-test between pairs of models, which further verified the observed performance differences. Therefore, the Custom CNN model is the most dependable and efficient in the field of gender classification.

### 5.6 Comparative study of models

Table 2 illustrates that CNN is the most effective performer in this comparison, primarily due to its superior balance of practicality, efficiency, and accuracy. It demonstrated its capacity to generalize effectively on the dataset by achieving the maximum values of Precision (0.90), Recall (0.91), F1-Score (0.905), and Accuracy (0.92) in the evaluation criteria. Additionally, the training and inference speed of CNN is sufficient to be implemented in real-world systems that require low latency and responsiveness. Although the computational cost is relatively low in the configuration under consideration, it is a top contender for synthesis on devices that lack adequate hardware support, such as mobile phones or embedded systems. Additionally, CNN is significantly more adaptable than fixed pre-trained models such as ResNet50 or VGG19, as it can be optimized specifically for this dataset due to its custom building.

**Table 2:** Summary of all models.

Criteria	CNN	ResNet50	VGG19
Model size	Small (~few MBs)	Large (~98 MB)	Very large (~143 MB)
Training time	Fast (~few mins)	Moderate (~10–15 min)	Slow (~15–20+ mins)
Inference speed	Fast (real-time)	Moderate	Slow
Complexity	Low (custom CNN)	High (deep residual blocks)	Very high (19 conv layers)
Overfitting tendency	Easy to control	Sometimes (needs tuning)	High risk (many parameters)
Flexibility	Easily customizable	Rigid pre-trained architecture	Harder to customize

### 5.7 Prediction

**Figure 16:**
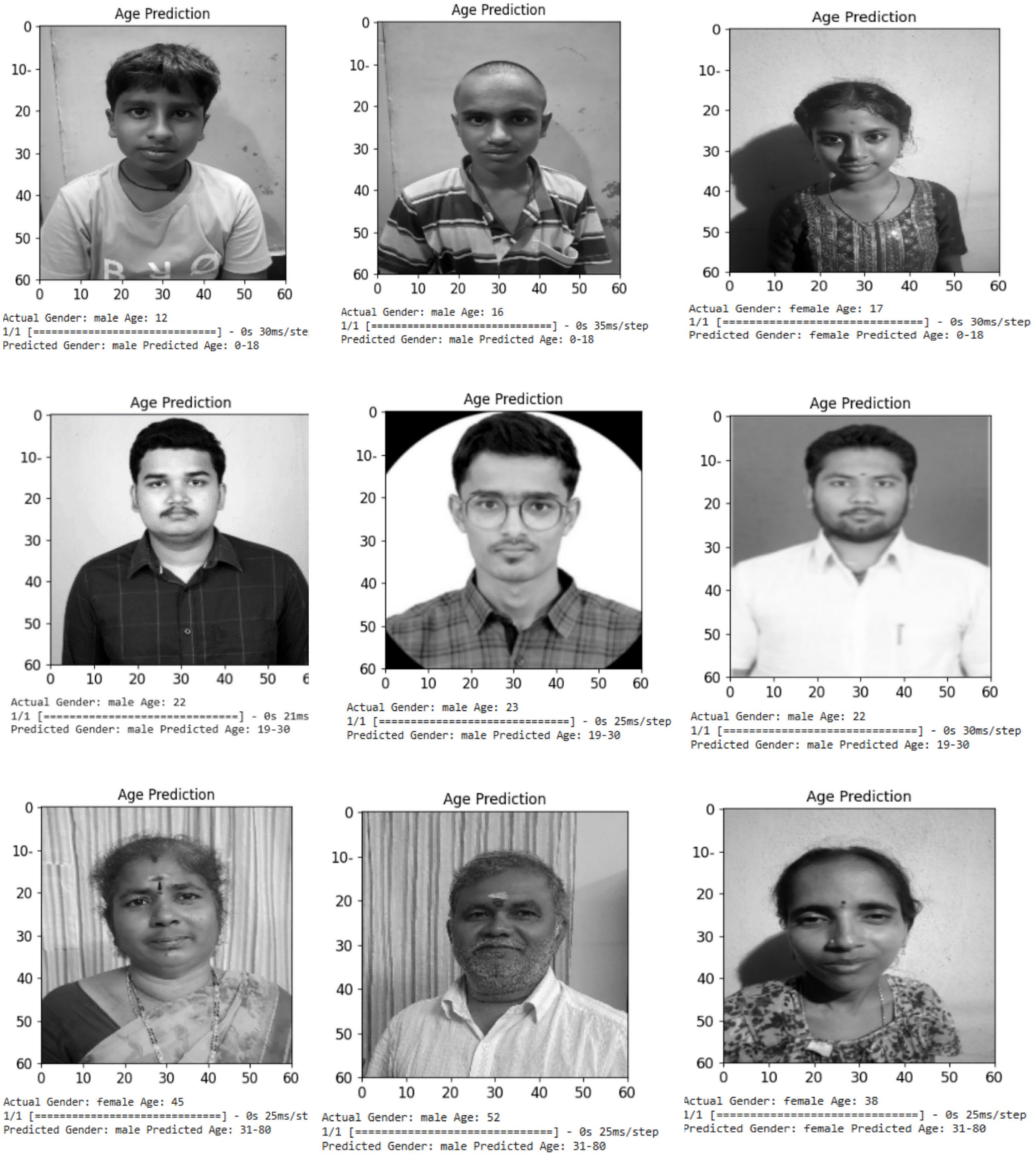
Simulation results of age and gender prediction of the uploaded sample images.

Figure 16 illustrates the simulated outcomes for the age and gender determination of the uploaded sample images. The CNN model was capable of accurately predicting the gender and age of the images that were submitted for validation.

### 5.8 User Interface

**Figure 17:**
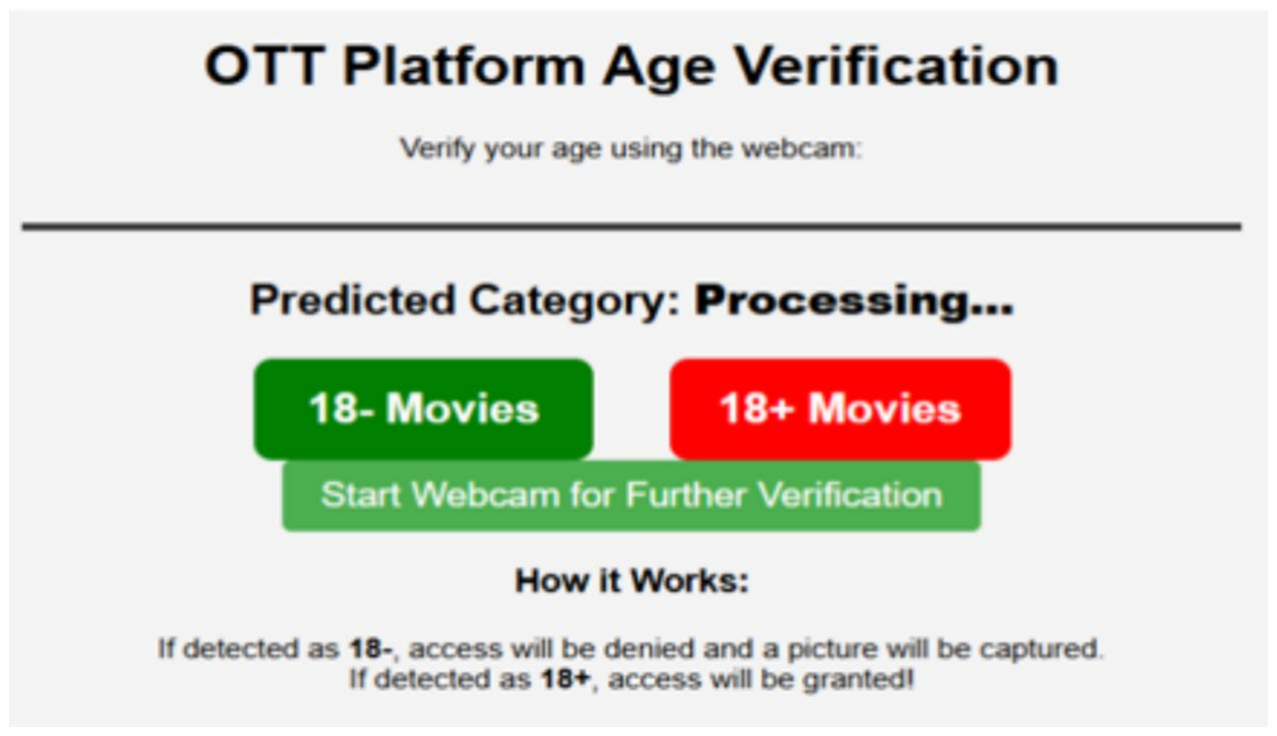
User interface for age verification.

An interface identified as “OTT Platform Age Verification” is illustrated in Figure 17. This interface utilizes the webcam to determine whether a user is eligible to view age-restricted content. It is displaying two categories of content, “18− Movies” and “18+ Movies,” and allows users to activate the webcam for additional verification. The system then predicts their respective ages based on indications from their appearance after processing begins. Access to the 18+ content will be denied if the user is under the age of 18, and their photo will be taken for monitoring purposes. However, access to age-restricted movies will be granted if the user is 18 or older.

The system design and performance specifications of the user interface that was devised for real-time age group prediction through webcam input are depicted in Table 3. HTML and CSS (Jinja2) were employed to develop the UI frontend, which is generally compatible with all browsers and renders rapidly (~0.5 s). Backend servers in Python that are based on Flask offer efficient routing with response times of 10–30 ms for PCs, laptops, and mobile devices. Model inference was conducted using an H5 model that was built on Keras, with prediction times of approximately 10–12 ms in the absence of GPU support. The video frames are being captured at a rate of 10–15 FPS on PCs and 5–8 FPS on mobile browsers using OpenCV. The prediction logic operates on straightforward Python conditions of classification into “0–18” or “18+” categories, necessitating a similar 1 ms. Browsers such as Chrome, Firefox, and Edge support frame streaming, which enables the transmission of video feeds with a delay of less than 100 ms on a local network. This is achieved through multipart HTTP (MJPEG). The security layer based on Flask allows for the real-time filtering of explicit content server-side for users over the age of 18, without compromising device compatibility or performance.

**Table 3:** System design and performance specifications for user-interface.

Component	Technology	Purpose	Performance	Device compatibility
UI frontend	HTML, CSS (Jinja2)	Display webcam feed, show predictions	Loads in ~0.5 s	All modern browsers
Backend server	Flask (Python)	Handle routes (/, /video_feed, etc.)	~10–30 ms response time	PC / Laptop / Mobile
Model inference	Keras (H5 model)	Predict age category from frame	~10–12 ms per prediction	Efficient on CPU, no GPU needed
Video capture	OpenCV (cv2)	frames from webcam	10–15 FPS on PC	Mobile browsers slower (~5–8 FPS)
Prediction logic	Python conditions	Decide “0–18” or “18+” group	~1 ms	Lightweight everywhere
Frame streaming	Multipart HTTP (MJPEG)	Continuously stream webcam to frontend	Smooth on LAN (<100 ms lag)	Chrome, Firefox, Edge
Security layer	Flask (server-side)	Restrict access to adult content (18+)	~Instant decision	Device agnostic

**Figure 18:**
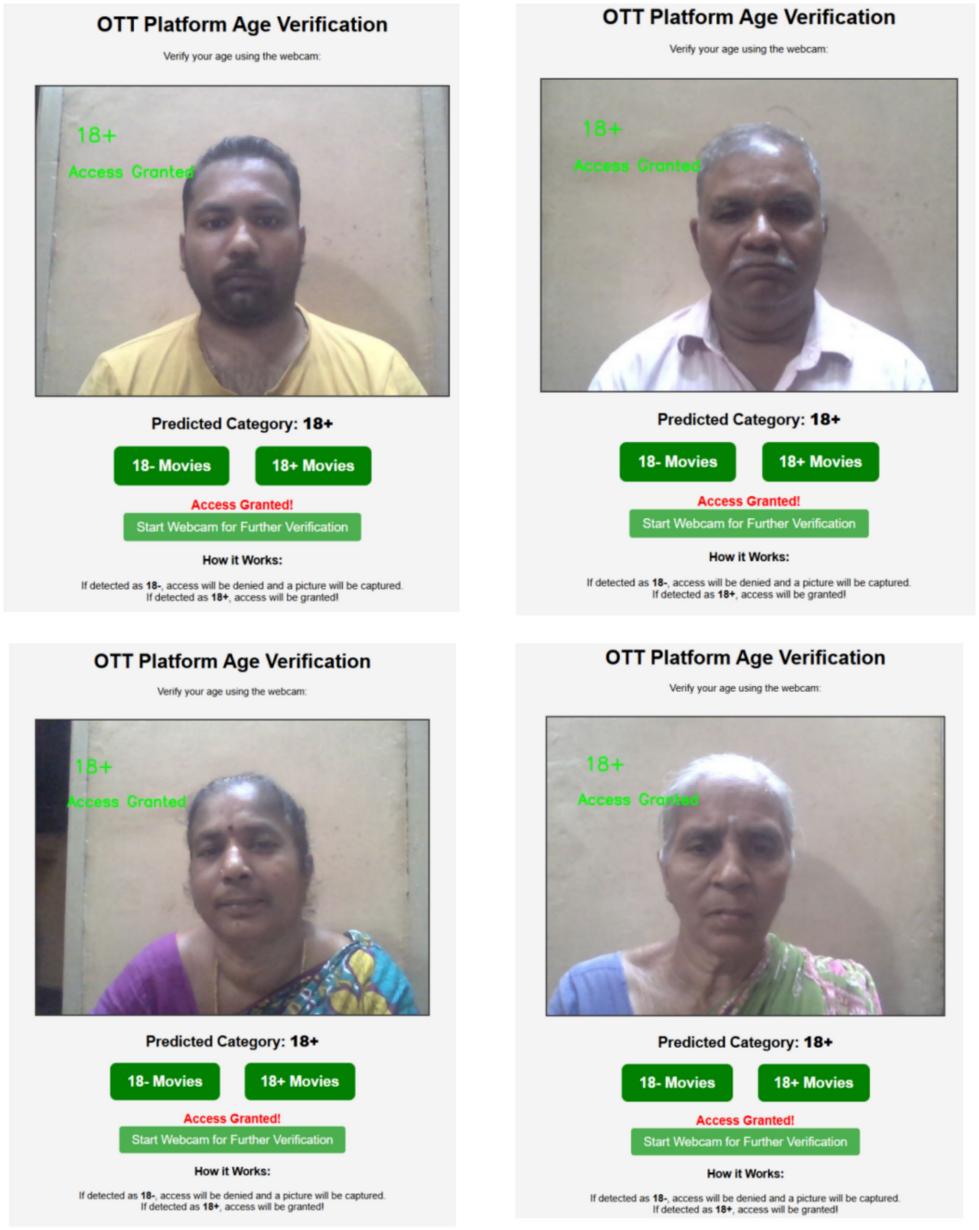
Access granted for 18+ videos.

The interface successfully authenticated the user’s age through facial recognition and designated them as “18+” to grant them access, as illustrated in Figure 18. It displays a webcam view of the user and the message “Access Granted,” indicating that the user is eligible to view age-restricted content. The interface offers the option to select the category for “18− Movies” or “18+ Movies,” and the verification result is plainly displayed as “Predicted Category: 18+.” Consequently, the user validation process is automated to anticipate the user’s level of control over access to content databases.

**Figure 19:**
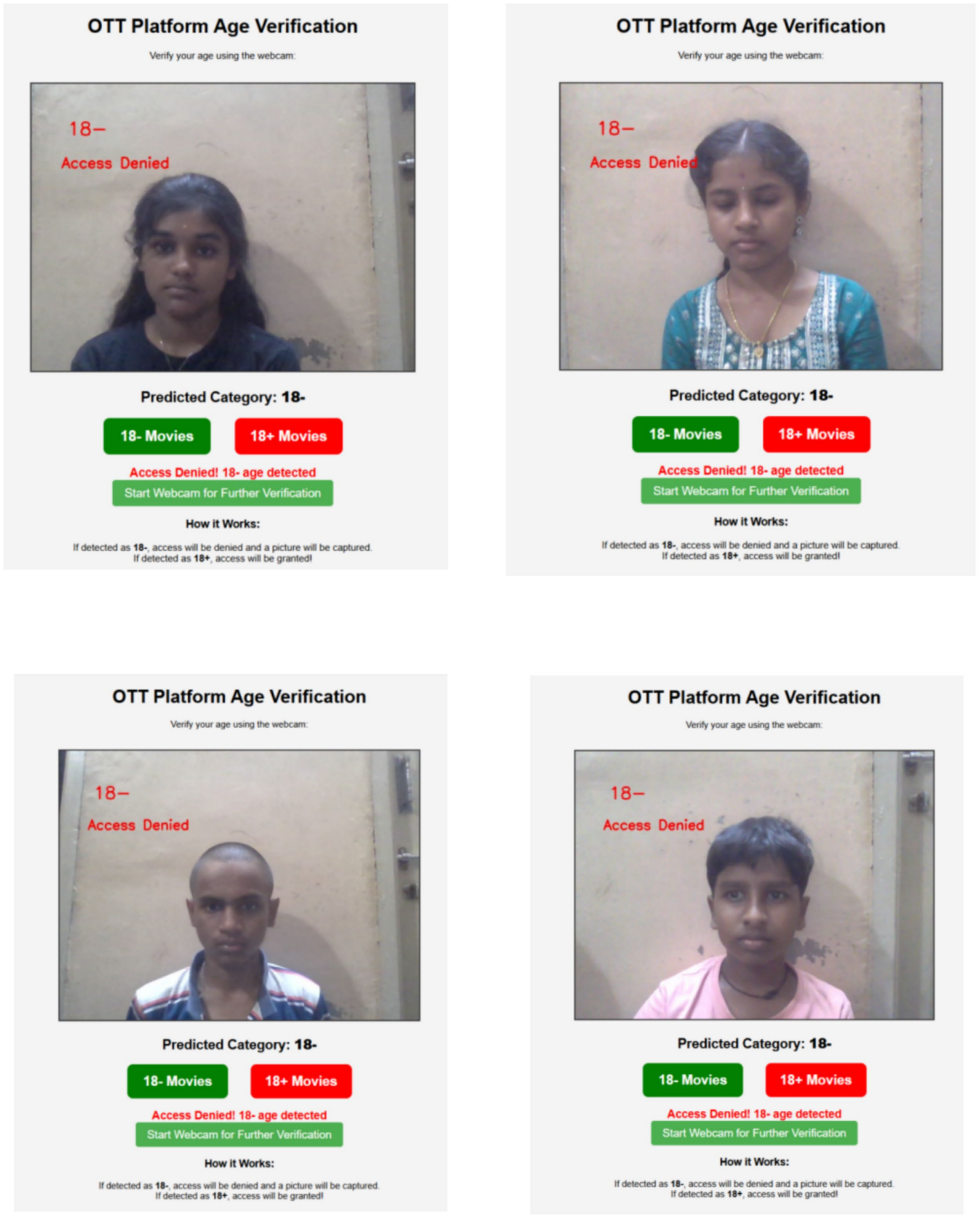
Access granted for 18− videos.

The age verification system of the OTT platform recognizes a user as being under 18 years of age in Figure 19. The interface displays the message “18– Access Denied” in red letters above the image captured from the webcam, preventing access to content that is inappropriate for the user’s age group. The predicted category below is “18–,” which means that users under the age of 18 are prohibited from accessing the “18+ Movies” and are only permitted to access the “18– Movies.” This mechanism is designed to ensure the responsible dissemination of content by enforcing age-based access control.

The current OTT age and gender verification system implementation does not save or capture any images, thereby ensuring that the user’s privacy is maintained in accordance with the default. Face counterfeit detection has not yet been implemented. In the future, it may be feasible to eliminate the requirement for image storage by employing OpenCV (cv2.imwrite) to capture images of access-denied attempts. In order to enable parental surveillance when children are accessing or attempting to access unwanted content, such images can be securely distributed to the account administrator. In this manner, user awareness and assent are honored, as it is customary to distribute credentials within families. Regardless of its implementation, all environments in which the stored data are located will be subject to privacy laws, such as the GDPR. This includes mechanisms for encrypting data and restricting access to ensure the integrity of the system and the privacy of users.

### 5.9 Comparison of existing work with the proposed framework

Existing research on age and gender estimation primarily focuses on improving classification accuracy using deep learning models evaluated on benchmark datasets which treat demographic prediction as an isolated computer vision task. Most prior works do not address real-time deployment challenges, platform integration, or automated content restriction mechanisms. However, our proposed system extends beyond traditional prediction-based approaches by integrating live camera-based verification within an OTT environment. OpenCV is used for facial detection, while Customized CNN, ResNet50, and VGG19 models are trained and evaluated independently for age and gender classification. Our proposed system provides a deployment-oriented application-driven framework which enhances child safety and responsible content consumption on OTT platforms whereas existing works focus only on model development.

## 6 Conclusion

The present study suggests a deep learning-based initiative for age and gender verification on a variety of OTT platforms. This is critically important in light of the very real issue of children accessing objectionable content using parental accounts. By emphasizing the importance of making the appropriate decision regarding whether a user is over 18 years of age, the authors promote responsible content consumption and alleviate parents’ concerns. In terms of age and gender-oriented classification, the Convolutional Neural Network (CNN) demonstrated the highest capacity to acquire complex features, with an accuracy of 91%, among the various models that were tested. ResNet50 has a balanced accuracy of 82%, which is a result of the residual connections that enhance learning without adding excessive complication to the model. The VGG19 model has a very low accuracy of 52%, which suggests that it may be susceptible to overfitting. As a result, this investigation not only prioritizes the protection of minor users but also fortifies our commitment to the development of a digital environment that is more conducive to the safety of users on OTT platforms.

A method will be employed by the user interface to determine the user’s age by measuring the camera video stream. Users are permitted to view the film provided that their age is equivalent to or greater than that of the film, as indicated by the user interface. The user interface for viewing the film is denied access if this is not the case. This proposed User Interface offers a practical method for OTT platforms to verify the age of their consumers. In the future, consumer responsibility could be improved by creating a tool that captures images of individuals who are attempting to access age-restricted content fraudulently. We will then forward the snapshot directly to the account holder for action, thereby confirming our dedication to responsible and safe content consumption on our OTT platform.

The proposed research highlights not only the technical feasibility of deep learning-based age and gender verification but also its broader social value in reinforcing digital child safety. By addressing both parental concerns and corporate responsibility, the proposed solution sets a foundation for more ethical and accountable OTT content delivery in the future.

The study investigates child safety on OTT platforms through its current complaints about technical performance which leads to responsible content consumption and better parental control solutions. The study assesses model performance for demographic prediction through comparisons between Customized CNN and ResNet50 and VGG19 models. The experimental study gains transparent and reproducible results because researchers use the publicly accessible UTKFace benchmark dataset.

## 7 Limitations and real-world deployment challenges

The CNN-based age and gender verification model achieves high testing accuracy which becomes disrupted when applied to actual OTT platform operations.

### 7.1 Lighting variations

The actual world experiences multiple lighting conditions which do not maintain consistent patterns. The system struggles to maintain accurate age estimation because various lighting conditions which include low light and overexposure and shadowing and colored lights and backlighting. The system requires to manage unpredictable light conditions which occur in actual use because benchmark datasets do not provide stable light conditions.

### 7.2 Occlusions (mask, hair, hand)

The process of extracting facial features becomes difficult when partial facial areas become blocked. Users can use masks to block their faces while they touch their face or block their forehead and cheeks with hair. These facial blocks prevent the system from seeing age features which results in wrong age estimate.

### 7.3 Pose variations

The model requires training with facial images which show near-frontal views of human faces. Yet users in actual environments show their faces through head tilts and side profiles and facial positions which lack alignment. The model performance decreases when people assume different body positions which lead to diminished facial symmetry.

### 7.4 Accessories (glasses, caps)

People who wear eyeglasses and sunglasses and caps and head coverings conceal critical facial identification points. The system maintains performance with minor accessories yet heavy facial blocks create age estimation errors.

### 7.5 Background clutter

Users can access OTT platforms through their actual deployment from locations that have challenging background conditions and extreme noise levels. Face detection accuracy and subsequent face cropping operations which lead to classification work are disrupted by background clutter and multiple faces within the same frame and the presence of moving objects.

### 7.6 Webcam quality differences

The implementation of the system on various devices leads to differences in hardware performance. The various webcam characteristics which include resolution and frame rate and motion blur and compression artifacts and network latency will create issues that result in decreased image quality which further impacts prediction accuracy.

#### 7.6.1 Face spoofing limitation

The system operates with live camera-based verification but lacks specific anti-spoofing and liveness detection systems. The system remains open to presentation attacks because attackers can use photographs and pre-recorded videos to trick the camera. The security of deployment will improve through the implementation of advanced liveness detection methods which include blink detection, motion analysis and depth-based verification.

#### 7.6.2 Dataset bias and fairness considerations

The proposed framework is trained and evaluated using the UTKFace dataset, a publicly available benchmark for age and gender estimation. The UTKFace dataset exhibits distributional biases which are typical to most real-world datasets because its age groups are not evenly distributed across various demographic categories. The model will learn different behaviors due to their age range and ethnic group representation which will cause problems in achieving fairness and generalizing results during actual implementation.

The training sample variability was increased through data augmentation methods while individual age groups and gender categories were evaluated through stratified K-fold and Monte Carlo cross-validation. The measures create evaluation standards that better protect against evaluation bias and increase testing strength, yet dataset bias remains impossible to completely remove. The upcoming research studies will use multiple external datasets to conduct validation tests which will evaluate three factors: fairness, reliability, and ability to generalize to real-world situations.

## Data Availability

The UKTFace dataset used in this research is publicly available at https://www.kaggle.com/datasets/jangedoo/utkface-new.
